# A Smart Sensing Technologies-Based Intelligent Healthcare System for Diabetes Patients

**DOI:** 10.3390/s23239558

**Published:** 2023-12-01

**Authors:** Sana Maqbool, Imran Sarwar Bajwa, Saba Maqbool, Shabana Ramzan, Muhammad Junaid Chishty

**Affiliations:** 1 Department of Computer Science, COMSATS University, Lahore 54000, Pakistan; sanamaqbool02@gmail.com; 2 Department of Computer Science, The Islamia University of Bahawalpur, Bahawalpur 63100, Pakistan; 3 Department of Computer Science & IT, GSCWU Bahawalpur, Bahawalpur 63100, Pakistan; shabana@gscwu.edu.pk; 4 Shaukat Khanam Memorial Cancer Hospital and Research Center, Lahore 54000, Pakistan; junaidchishty@skm.org.pk

**Keywords:** human-centered application, IoT, machine learning, smart healthcare prediction, fuzzy logic, decision support system

## Abstract

An Artificial Intelligence (AI)-enabled human-centered smart healthcare monitoring system can be useful in life saving, specifically for diabetes patients. Diabetes and heart patients need real-time and remote monitoring and recommendation-based medical assistance. Such human-centered smart healthcare systems can not only provide continuous medical assistance to diabetes patients but can also reduce overall medical expenses. In the last decade, machine learning has been successfully implemented to design more accurate and precise medical applications. In this paper, a smart sensing technologies-based architecture is proposed that uses AI and the Internet of Things (IoT) for continuous monitoring and health assistance for diabetes patients. The designed system senses various health parameters, such as blood pressure, blood oxygen, blood glucose (non-invasively), body temperature, and pulse rate, using a wrist band. We also designed a non-invasive blood sugar sensor using a near-infrared (NIR) sensor. The proposed system can predict the patient’s health condition, which is evaluated by a set of machine learning algorithms with the support of a fuzzy logic decision-making system. The designed system was validated on a large data set of 50 diabetes patients. The results of the simulation manifest that the random forest classifier gives the highest accuracy in comparison to other machine learning algorithms. The system predicts the patient’s condition accurately and sends it to the doctor’s portal.

## 1. Introduction

The Internet of Things (IoT) and machine learning (ML) are two rapidly evolving technologies that have profoundly impacted the healthcare section [1]. The convergence of these technologies has given rise to innovative and smart solutions in the form of IoT-based human-centered healthcare applications utilizing ML [2]. IoT-based healthcare applications for portable and remote monitoring with a focus on human-centricity are needed by those who live in underdeveloped or remote areas. Diseases like high blood pressure, heart disease, and diabetes are long-lasting and require continuous monitoring [3,4].

In IoT-based applications, sensors enable real-time data collection from diabetic patients, providing a continuous stream of vital signs and other health-related information. This constant monitoring ensures a comprehensive view of the patient’s health status. This vast amount of data is processed via ML to identify patterns, anomalies, and trends, assisting in personalized diagnosis and treatment plans. It can predict disease conditions and evaluate factors, on the basis of which risk assessments can be made for personalized interventions [5]. ML algorithms heavily rely on the quality and quantity of data. Existing data on diabetic patients are for patients who are demographically different and have different levels of health-service challenges as compared to local-area patients. Thus, inherent biases exist due to diverse demographic and environmental factors that affect human health. Inadequate or biased data may lead to incorrect predictions and recommendations [4,6]. According to the best of our knowledge, the specific data set related to diabetic patients in Pakistan is limited as compared to the data sets of other countries. Looking for data on diabetic patients in Pakistan, we need to contact healthcare institutions, hospitals, research organizations, researchers, and government health departments. We cannot gain access to specific sensor data through our contacts and by meeting with them. Thus, we decided to design and develop an IoT system for data collection for local patients for the betterment of Pakistan’s underdeveloped areas like [7,8]. When accessing and using healthcare data, especially patient-related data, ethical and legal guidelines must be followed. Data privacy and consent are critical considerations when working with sensitive health information.

Our whole work focused on smart health applications rather than simple health applications. A comparison between the pillars of smart health applications and simple health applications and their implementation is essential to understand the differences and similarities between these two approaches to healthcare technology, as described in Table 1 and Table 2. 

**Figure 1:**
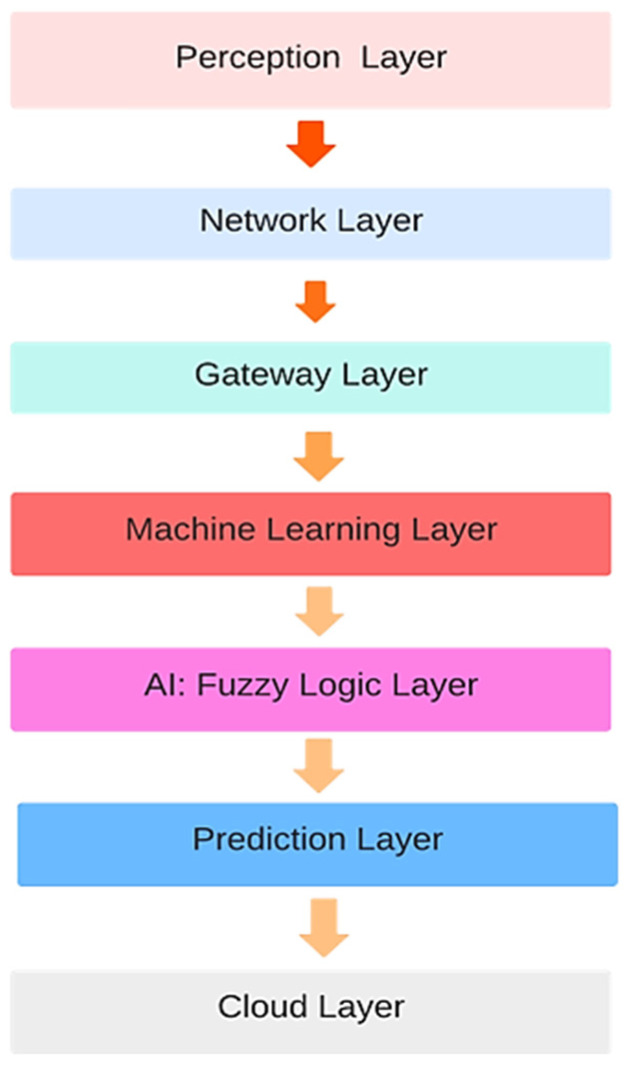
Layered architecture of the designed system.

Smart health applications involve some advanced technologies like ML and AI (Table 2). Remote health monitoring with privacy and security concerns under the supervision of professionals is also a feature of smart health. All these smart health features of Table 2 are depicted in our layered architecture in Figure 1 and also in our component-based architecture in methodology section.

IoT devices facilitate personalized care by tailoring interventions based on individual health data. Patients receive custom alerts, recommendations, and treatment plans, enhancing engagement and outcomes. Customization may require sophisticated and resource-intensive IoT infrastructure, potentially increasing the overall cost of healthcare services. ML enhances personalization by analyzing data to create precise models for each diabetic patient. Like ML, decision support systems in human-centered IoT devices with healthcare applications can trigger real-time alerts for healthcare professionals or caregivers in case of emergencies or abnormal health parameters. This enables timely interventions and improved patient safety. ML algorithms can provide automated decision support systems by analyzing incoming data and suggesting appropriate actions based on established patterns and medical guidelines, for instance, in diseases affecting factors like normal, high, and low ranges of vital signals, especially blood glucose levels [10,16,17,18].

Bandyopadhyay et al. [19] discuss the fact that accessibility and scalability are challenges for increasing numbers of patients when it comes to dealing with large amounts of data during continuous remote monitoring. IoT solutions can be scalable to accommodate a large number of users and devices [8,17]. Diabetic patients need to check their glucose levels after and before every meal and take doses accordingly. Cloud-based architectures facilitate easy accessibility and data sharing across healthcare systems.

Considering all these points, a smart and intelligent decision-making system is presented here for diabetes patients. The proposed smart health architecture consists of five sensors for the measurement of health conditions, namely, temperature, heart rate, oxygen level, blood pressure, and blood glucose level via non-invasive measurement through an infrared (IR) sensor. A gateway node, the Arduino Nano, is used that collects all the data through sensors and sends them to an online server through Wi-Fi connectivity. 

Secondly, we propose a smart algorithm to effectively send data to a server that gives priority to abnormal ranges of data, which can eventually lead to unhealthy and critical condition prediction by the ML algorithms that are applied so that doctors can effectively deal with emergency patients, prioritizing them over patients whose body vital measurements, like BP, heart rate, temperature, blood glucose level, and oxygen saturation, are in normal ranges. 

Thirdly, we have designed a hardware circuit for the non-invasive measurement of blood glucose levels, as glucometers available in underdeveloped areas are dependent on invasive methods of measurement. Frequent penetration of needles for continuous monitoring is painful and skin-damaging. The proposed glucose sensor for diabetic patients is a wearable sensor on the thumb, and there is only a need for light penetration and keeping the thumb in a direction such that the LED light does not face the nail portion of the thumb. The details of this sensor are discussed in the proposed methodology and architecture section.

In addition to this, we validate our proposed smart wearable healthcare system architecture and its measurements with standard apparatus measurements that are already available in hospitals.

Moreover, our system also predicts a patient’s current health condition based on the sensor data measures collectively using ML algorithms. We use support vector, Gaussian Naïve Bayes, decision tree classifier, random forest, and Bernoulli NB and ML techniques for the prediction of health conditions, including healthy, moderately unhealthy, unhealthy, very unhealthy, critical, and very critical, based on classified sensed data. For accurate health condition predictions, appropriate rules and fuzzy sets are needed; thus, using fuzzy logic, we developed a fuzzy logic-based decision support system for making decisions about patient’s health conditions. We achieved results in the form of accuracy, precision, recall, and F1 scores for our proposed system’s patient records and also for the standard apparatus’ records and compared them. 

When designing a healthcare system using non-invasive glucose measurement and performing clinical procedures with NIR light, it is important to make certain assumptions to simplify the model and provide a basis for its development and performance evaluation. Here are some assumptions we made:

- It was assumed that patients do not have serious skin diseases that cause redness or itching due to light absorption.
- It was assumed that patients will not have applied any medicine or compound on a skin area that faces the hardware, such that measurements will not be affected.

To address the skin issues in a clinical scenario, users and healthcare professionals have to be educated about the system’s limitations and the best practices for using the device in patients with dermatological conditions. Proper sensor placement and skin preparation help in mitigating some challenges [20,21].

The rest of the paper is organized as follows. Section 2 discusses related work. Section 3 presents the proposed architecture and the work methodology. Section 4 presents the results and discussion. Finally, conclusions and future work are outlined in Section 5.

## 2. Related Work 

Diabetes is a chronic disease or group of metabolic disorders. Blood glucose levels in the body are elevated, which is either due to a lack of insulin production or high sugar levels in the circulatory system [18]. This is due to malfunction of the pancreatic beta cells. It affects different parts of the body, and risks include pancreatic disease, heart disease, hypertension, kidney failure, pancreatic problems, nerve damage, foot problems, ketoacidosis, visual impairment, and many more. Eye problems, waterfalls, and glaucoma are also potential risks. There are various reasons behind the causes, such as lifestyle, lack of activity, weight gain, smoking, high cholesterol (hyperlipidemia), high blood pressure (hyperglycemia), and others, which can be targeted to treat diabetes. It affects various age groups, from adolescence to adulthood. The pancreas is an organ located in the midriff area. Gangil et al. [15] proposed a strategy that uses an ML technique, namely, an SVM, for classification for diabetes analysis. The ML strategy for diabetes management is based on a high-dimensional therapeutic data set. The experiment proved that the vector machine could be used to effectively diagnose diabetes.

Jara et al. [10] discussed smart sensing technologies that provide foundational data collection capabilities for IoT applications. Boursianis et al. [2] described the varieties of smart monitoring applications that have revolutionized several services not only in healthcare but in many other domains, like agriculture, networks, traffic control, and education, and in emergencies like fire disasters [22]. Challoner et al. [16] described smart healthcare services and digital health diagnosis performed using IoT. Alturjman et al. [4] discussed the authentication, integrity, and privacy of data using WSN in IoT. Rghioui et al. considered [23] continuous monitoring of patients using an IoT embedded system that makes health predictions based on machine learning algorithms, where an SMO algorithm gave the best results. Its results were evaluated in terms of accuracy, precision, and sensitivity. The IoT plays a dynamic role in the integration of physical and virtual items, as stated by Kavithamani et al. [1], who also discussed a health monitoring system based on IoT. IoT is crucial for developing cutting-edge technologies like wireless sensor networks, AI, cloud computing, robotics, transportation, and healthcare systems. According to the authors of [1,24], healthcare systems have seen many improvements, but glucose monitoring systems are still expensive and have several other drawbacks. The authors discuss the design of a glucose monitoring system based on IoT sensor devices in this work. The system can monitor real-time glucose levels, body temperature, and contextual data and give them to doctors and patients in a graphical and understandable format. Additionally, this model added push notifications to alerts.

Smart healthcare or e-health plays a vital role in treatment and smart health prediction in diabetes. With the integration of technology and data-driven approaches, healthcare providers can offer more personalized and efficient care to diabetes patients [25]. Islam et al. [26] introduced the development of a smart healthcare monitoring system in an IoT environment. Five sensors are used: a heartbeat sensor, a body temperature sensor, a room temperature sensor, a CO sensor, and a CO_2_ sensor. The patient readings are conveyed to the doctor’s portal and the doctor will give recommendations accordingly. The hardware they used was an ESP32, a heartbeat sensor, a temperature sensor, a CO sensor, and a CO_2_ sensor. The architecture of this paper involves the collection of data from the sensors that are received by the ESP32, and then these measurements are transferred to a web server which is connected to the user interface of medical staff. The implementation details involve actual readings compared with observed readings for their proposed system [18]. We also followed this basic architecture of data collection, sending them to the gateway, and then to a server for doctors’ access, additionally giving ML support for data classification. To demonstrate the novelty of the work, we also needed to give collective results for all classes and thus used fuzzy logic for making rules and finally predicting the patient’s health condition accurately [27]. The predicted health condition is a predicted doctor’s decision which can be analyzed by the doctor, and they can give recommendations based on it. The system helps make doctors’ decisions smarter.

Rghioui et al. [23] worked on a smart architecture for diabetic patient monitoring using ML algorithms. They monitored blood sugar levels, temperature, and physical activity using portable sensors and collected data. They collected data day-wise, in the morning, afternoon, and evening, in addition to the no. of steps taken in a day. The data are classified with a number of ML algorithms and compared, and the performance results are transferred to the doctor for deciding on the patient’s health.

Chatrati et al. [28] presented a smart home health monitoring system for predicting type 2 diabetes and hypertension. For these two diseases, they collected readings of blood pressure and blood glucose levels. For the prediction of status, they used ML algorithms and then gave notifications of status. The traditional approach was used; no rules were defined in it. The status representation is in the form of a category they found.

Qureshi et al. [29] presented an accurate and dynamic predictive model for a smart M-Health system using machine learning which they used to collect data through mobile applications. They proposed a secure Android application and reliable data storage and then transferred data for further ML processing. By means of ML algorithms, they classified cardiovascular diseases according to the seriousness of the conditions. They split the data into four folds, and predictive model decision trees and an SVM are used for prediction analysis. The performance of the predictive model with the benchmark was obtained in the form of accuracy, precision, and sensitivity.

Istepanian et al. [7] focused on the diagnosis of diabetes patients, and Rghioui et al. [23] worked on 5G technology, an invasive blood glucose measurement technique, and data classification utilizing ML algorithms. The data collection of sensors and smart devices like microcontrollers that receive data signals from sensors and deliver them to a server over Wi-Fi was also covered in this article. Similarly, Kavithmani et al. [1] employed NIR and continuous monitoring for diabetic patients to measure blood glucose levels. Photoplethysmography was used in this article for light with wavelengths of 525 nm, 660 nm, and 950 nm. Additionally, the authors contrasted the data gathered using the suggested system method versus conventional techniques. Rghioui et al. [23], suggested an IoT system for tracking health, and they also provided an alarm system for examination and analysis by doctors. They also made use of sensors for blood pressure, temperature, respiration rate, and heart rate, and data were collected through a gateway MCU (microcontroller unit). As a result, continuous health monitoring systems are becoming more and more developed over time as a result of the present need to stop the spread of diseases and reduce the death rate. A smart monitor study also suggested an IoT healthcare system that works on two sets of data connected to physiological activity and uses advanced electronic instruments for signal measurement like myRIO [30]. To separate features from a variety of sensory data, they presented a deep neural network. There has been a lot of effort and research on medical IoT in recent years, covering a variety of topics. One illustration is the use of mobile devices in the control of diabetes. To enable their services to be completed in the future, new technologies are being implemented, such as the connection of IPV6 and 6loWPAN in fourth-generation networks [10]. Rghiui et al. [23], in comparison to data gathering in non-real-time environments without temporal accuracy, adopted this technology for glucose monitoring, allowing clinicians to obtain accurate data over a given period [11]. The system uses non-invasive sensors to measure glucose levels. By using the opto-physiological model, such measurements are attainable. 

There are diverse healthcare systems for diseases like blood pressure, hypertension, and diabetes [25]. These diseases require long-run continuous monitoring; thus, there is a need for portable smart healthcare systems that gather sensitive data from the patient’s body and transfer it to the relevant physician.

Abrar et al. [5] focused on hypertension as a problem for health since it can cause several chronic heart conditions, including stroke, renal failure, and cardiac arrest. Multiple risk factors, including a person’s lifestyle and genetics, can result in hypertension. For doctors, it is challenging to stop certain illnesses developing early. Although there have been many ML-based solutions suggested, none of them has been tailored for the diagnosis of hypertension. Existing models are not updated with fresh data. Since there is no method for identifying anomalies in data, the outcomes are unpredictable. This research introduced a multi-agent system that can compute missing values concerning time and provide personalized prediction of the risk of hypertension. Abrar et al. [5] obtained real-time blood pressure readings using a Gaussian process model. The new input data are also learned by this model. The Framingham estimator, which can compute data over four years, was used to estimate the risk of hypertension. Blood pressure data for the Malaysian population were used to calculate the mean absolute error, the root mean square error, and the mean square error. The proposed multi-agent model was more accurate at making predictions than the current prediction techniques. A framework that utilizes a multi-agent approach was suggested in this paper. The development of a personalized system that can forecast the possibility of hypertension was the aim of this framework. This technology collects blood pressure readings at regular intervals and evaluates the results 24 h later. It foretells the likelihood of hypertension. The multi-agent-based methodology was used for that system. This method allows for the division of the work among four separate agents, each of which carries out a specific mission.

Munir et al. [31] described an intelligent and smart watering system that uses fuzzy logic and blockchaining for correct decisions about the consumption of water in small gardens. Thus, fuzzy logic is a supportive technique for decision making in smart systems. CR-based smart healthcare systems using tree-based ML algorithms were used in [32]. Through this article, we learn that ML is helpful and playing a role in healthcare systems. Here, we propose a patient monitoring system for diabetic patients that involves changes to the architecture described in this article in terms of the sensors used and the inclusion of a smart algorithm, an AI and ML hybrid approach being used in our proposal. As our architecture for transferring data from the sensor to the application was also layered, Table 3 provides a comparative analysis of IoT systems that employ a layered architecture and sensing parameters for data collection.

Sarwar et al. [27] worked on a fire detection system using an adaptive neuro fuzzy inference system. True detection of likelihood of fire is a novelty of their work. The implementation was performed in MATLAB. This system uses smart sensing technology for fire detection.

Afreen et al. [33] worked on an IoT-based monitoring and notification system using predictive analysis via an artificial neural network. Fruits and vegetables play an important role in minimizing the impact of some diseases; thus, the work focused on cold storage. The factors analyzed by the monitoring system were gauge temperature, relative humidity, and other vital ambient environmental parameters, such as luminosity and the concentration of gases. 

Shafi et al. [12] also utilized IoT and machine learning for an agricultural storage combustion system. Cotton storage has a number of limitations and challenges, i.e., heat due to microbial growth, exothermic and endothermic reactions in storage areas, and extreme weather conditions in storage areas. Monitoring and real-time sensing predictions are performed by an artificial network and machine learning to control sudden change and to avoid damage to the quality of cotton. Saifullah et al. [34] also introduced a smart sensing system for radiation monitoring and a warning system. Radiofrequency electromagnetic rays have a significant impact on the human body; thus, using machine learning, various levels of EMR intensities are analyzed.

**Table 3:** Comparison between related work and proposed model.

System Name	No. of Parameters	Sensing Parameters Monitored	Health Condition Checked	Communication Protocol	No. of Layers	Technology Used
A personalized healthcare Monitoring system for diabetic… [25]	4	Glucose, heart rate, activity, temperature	Diabetes, general health	Bluetooth, Wi-Fi	5	Mobile app, wearables
A real-time health monitoring system… [35]	3	Heart rate, blood pressure, temperature	Cardiovascular	Wi-Fi	4	Wearables, IoT, Zephyr BT
IoT-based personal health care monitoring device for diabetic patients [36]	2	Glucose, ketones	Diabetes	Wi-Fi	3	Mobile app, cloud
Wearable IoT enabled real-time health monitoring system [30]	3	Heartbeat, temperature, blood pressure	Diabetes, cardiovascular disease, obesity	Wi-Fi	3	Mobile app, cloud
Proposed article	5	Blood glucose, temperature, heart rate, blood pressure, oxygen saturation	Diabetes, general health	Wi-Fi	6	Wearable sensor, cloud

There were three articles found on IoT-based human-centered healthcare systems. A comparative analysis of key features of these three contributions is shown in Table 4. The key features discussed for comparative analysis are the ML algorithms used in each article; the data collection approach is smart sensing for remote monitoring with wearable sensors. Healthcare services can be handled at a personalized level, but their level, medium or high, varies according to the article. Smart data processing by algorithms and decision support are key features that are not introduced as compared to our proposed work.

Hameed et al. [32] studied an intelligent IoT-based healthcare system developed using a fuzzy neural network. This study presents a cloud-based framework utilizing sensor and UCI repository data. The nearest neighbor (NN) classifier is preferred in it for disease identification. The architecture is also different from ours, and, in comparison to these authors’ work, we used ML algorithms for correct predictions [21,37].

## 3. Proposed Architecture and Methodology

### 3.1. Layered Architecture

In our smart healthcare wearable system, we propose a seven-layer architecture including a perception layer, a network layer, a gateway layer, an ML layer, an AI-assistance decision support layer, and a cloud layer, as represented in Figure 1.

- Perception Layer: This layer represents the interface between the physical world and the digital system. It comprises sensors, wearables, and other data acquisition devices. This layer collects data from various sensors (health monitors) and preprocesses raw sensor data to extract meaningful information. Data filtering filters out noise and irrelevant data to improve data quality.
- Network Layer: This layer focuses on data communication and management. It ensures seamless data transmission between the perception and gateway layers. This layer transmits preprocessed data from the perception layer to the gateway layer. It also utilizes network protocols for efficient data transmission.

The challenge with network layer is to ensure medical device safety against cyber-attacks or external interferences. Designing a communication architecture in a server like Thing Speak to ensure medical device safety against cyber-attacks or external interference is crucial for healthcare applications. For our proposed system, we used Thing Speak to store IoT data. Thing Speak follows specific design aspects to enhance medical device safety.

- Gateway Layer: This layer acts as an intermediary between the edge devices and the cloud server. This server has strict access controls and user authentication mechanisms. Only authorized users have access to IoT device data. The HTTPS secure protocol, which resists any interception or intrusion of data during transmission from IoT devices, is used. API keys are used to secure data and to control access [38]. This ensures device authentication and thus authorizes and secures data effectively. The system on which we are accessing data is firewalled, and intrusion detection that does not allow any intrusion is used. Data stored on Thing Speak can be protected. Other protection measures can be applied, but we are not using them currently for our data protection.

Gateway layer processes and filters the incoming data before forwarding it to the cloud. It aggregates and organizes data received from multiple devices for efficient processing. This layer conducts preliminary analytics and data preprocessing to reduce the data load on the cloud. Here, we have applied a smart algorithm to prioritize the emergency data of those patients whose sensor readings are abnormal such that they require urgent healthcare service. This layer converts data to a format compatible with cloud-based processing.

- ML Layer+ (AI) Layer: In this layer, collected data are loaded and, after this, data preprocessing and anomaly handling is performed. As there may be inadequate values, for instance, temperature values, which cannot be 0, 3, or 7, anomalies are removed in this layer. After preprocessing, understanding and feature extraction of the data is performed, and classes of data in different ranges are made. These classes are used as an input to the AI layer and fuzzy logic decision support for making rules and crisp decisions is applied. After decision support from the fuzzy logic, the layer trains ML models on real-time data to learn patterns and identifies unusual patterns or outliers in the data that may indicate potential health issues. It also predicts the likelihood of certain diseases or health conditions based on input data.

Our reasons for choosing fuzzy logic to help in classifying data and making decisions are as follows:

- Fuzzy logic is well-suited for situations where data are uncertain and vague. It allows for a clearer understanding of data and can handle cases where traditional binary logic might fail. Fuzzy logic enables a finer level of granularity in decision making. Instead of “yes” or “no” decisions, it allows for more options along a spectrum. This is particularly useful in healthcare, where patients’ health parameters can have varying degrees of severity.
- Fuzzy logic uses linguistic terms and rules that are more intuitive and human-centric. This makes it easier for healthcare professionals and patients to understand and trust the decision-making process. Fuzzy logic-based systems can adapt and learn from data over time. This is important in healthcare, as patient conditions and responses to treatments can change.
- Prediction Layer: This layer is responsible for making predictions and generating actionable insights based on the results obtained from the ML layer. It also provides predictions regarding a patient’s health condition, potential risks, and unhealthy conditions. This layer offers treatment suggestions and personalized information on a patient’s current condition which a doctor can use to take further measures and recommendations based on the ML model outputs. It also generates alerts for healthcare professionals or patients based on critical health indicators or predicted events.
- Cloud Layer: The cloud layer is the centralized computing and storage infrastructure where data are stored, processed, and analyzed. This layer stores vast amounts of historical and real-time data securely. The continuous monitoring of diabetic patients requires continuous uploading of data, for which a storage and access layer is required. Planning for rollback or reverting to a previous state is helpful in managing updates and the security of data. User education regarding updates and security patches means that users can be informed regarding any changes and updates [39]. Regular backups can be performed by downloading data and uploading them to a cloud for their recovery in case of any vulnerability or security issue.

**Figure 2:**
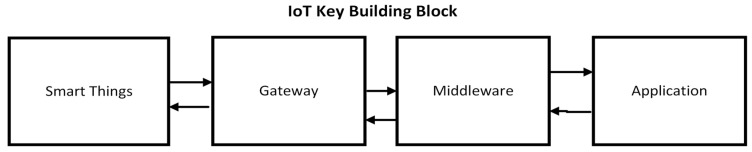
IoT basic building blocks [30].

The basic building blocks of the IoT layered architecture consist of four primitive layers Figure 2: a sensor layer, a network layer, a middleware layer for data storage and processing services, and, fourthly, an application layer, according to the domain and sensors used. Figure 1 and Figure 2 provide a comparison of the layers of the proposed work and the key IoT building blocks. In the proposed work, the ML and AI layers are added with a cloud support layer for continuously updating our patients’ data.

### 3.2. Proposed Architecture of the Smart Decision Support System

**Figure 3:**
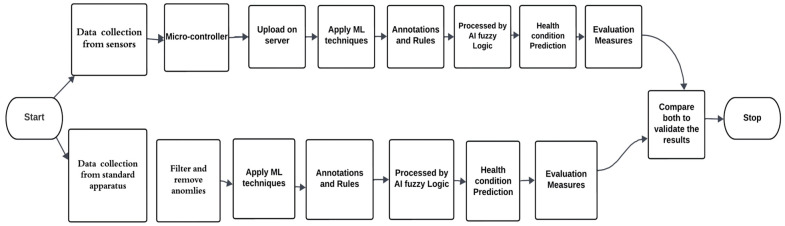
Flowchart of proposed work with system benchmarking.

Before the detailed description of the working methodology and component-based architectural details and after discussing the layers involved in our proposed work, these layers are repeated for data collected from standard apparatus for data validation purposes. Figure 3 represents the workflow followed for the proposed methodology validation. The work methodology for sensor data is synchronized according to the layered architecture, but the workflow for data collected from the standard apparatus first involves the organization of data on all vital signs for preprocessing and removal of anomalies. Then, data are loaded for the ML process, where preprocessing is again performed automatically by checking for missing and zero values. A similar feature extraction step to that used for sensed data is performed. Ranges of data sets are used as an input for the fuzzy logic decision support system. Fuzzy logic converts these fuzzy sets into linguistic variables, and membership functions and inference engines then give crisp values for health conditions which are compared according to the proposed methodology steps through different evaluation measures like accuracy, F1, and precision scores.

**Figure 4:**
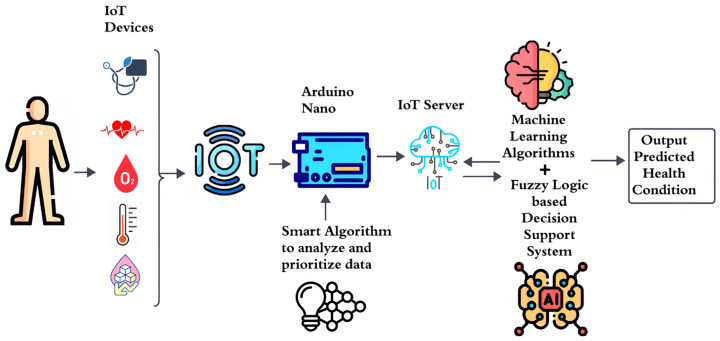
Architecture of smart decision-making support system.

The smart healthcare system comprises 5 sensors for taking health measures of diabetic patients. The sensors involved are temperature, blood pressure, oxygen level, heart rate, and sugar level sensors, as shown in Figure 4. These human-centered IoT devices are wearable on the hand and thus transfer data signals to the Arduino Nano microcontroller, in which we have applied a smart adaptive algorithm that analyzes the sensed data provided to it by limited normal health ranges through an array data structure and by analyzing personalized reading data packets sent on priority bases. Thus, emergency data indicating critical and very critical conditions are prioritized and give alerts to diabetic patients with beeps. In remote monitoring for long-term diseases, there is a need to give alerts and send data efficiently so that a patient can utilize intended services like a physical presence in the hospital.

The smart decision-making system, the following steps need to be performed:

- Data collection from sensors attached to the diabetic patient’s human body. Temporal changes in health condition can be overlooked by continuous monitoring of data and data variation patterns. The doctor can view patterns and graphs on the server that indicate gradual changes in health and previous health statuses predicted by the system. This can minimize the effect of temporal changes and support decisions regarding medication and dosage changes.
- The Arduino Nano is used as a microcontroller that receives the sensed data.
- A smart priority algorithm is applied to sensed data to give priority to emergency data of a patient so that emergency and critical patients can receive doctor’s recommendations effectively.
- Data are uploaded to the IoT server, as the data for continuous monitoring exist in large amounts.
- Data preprocessing and removal of inconsistent and missing data from stored data are performed to mitigate issues.
- For diabetic patient health condition predictions, we applied 5 ML algorithms for current health condition prediction.
- In designing a correct rule base and prepositions, we applied AI using fuzzy logic.
- The patient’s condition is predicted, and the prediction is sent to the relevant practitioner for further suggestions.

### 3.3. Fuzzy Logic-Based Decision-Support Smart Healthcare System

Fuzzy logic, a mathematical framework and computing paradigm, addresses uncertainty and imprecision in decision making and system control. It was introduced as an extension of classical (or “crisp”) logic to model and handle uncertainty, undefined concepts, and ambiguous or incomplete information. In classical logic, propositions are either true or false (binary), and there is no room for intermediate or partial truth values. Fuzzy logic, on the other hand, allows for a gradual transition between true and false, assigning a degree of membership between 0 and 1 to a proposition, representing the “fuzziness” or uncertainty associated with it.

#### 3.3.1. Fuzzification

Fuzzy sets are a generalization of classical sets, where an element can belong to a set to a certain degree, represented by a membership function. Membership values range from 0 (not a member) to 1 (fully a member). Considering the problem and understanding data classes and defined ranges of data variables like blood pressure, heart rate, glucose measure, oxygen level, and temperature, we use fuzzy logic for removing uncertainties and to obtain a defined rule base to determine a crisp state. Thus, in the ML process, model training is performed based on smart inference, and the evaluation base is defined by fuzzy logic. For fuzzification, the inputs of different data ranges and linguistic variables assigned to these data ranges. Linguistic variables for blood pressure: (low, normal, elevated, high)

- Linguistic variables for temperature: (low, normal, high)
- Linguistic variables for glucose level: (low, normal, elevated, high)
- Linguistic variables for heart rate: (low, normal, high)
- Linguistic variables for oxygen saturation (critical, worrisome, normal)

a - Blood pressure

For defining the membership functions for blood pressure categories (low, normal, elevated, and high), we use trapezoidal membership functions to represent the gradual transition between categories. Trapezoidal membership functions are defined by four parameters: ‘a’, ‘b’, ‘c’, and ‘d’, where ‘a’ and ‘d’ represent the start and end points of the plateau, while ‘b’ and ‘c’ denote the points where the membership function initiates its ascent and descent, respectively. In the following, trapezoidal membership functions are defined for the specified blood pressure categories considering the data ranges used after data analysis of blood pressure: 0–90, low; 91–119, normal; 120–129, elevated; and 130, high. These ranges are defined based on feature extraction from data, expert opinions, and research analysis of feature ranges for adults in the age group 22 to 60, as these factor ranges differ for the results and old-age people.

- Low blood pressure: function parameters: (a = 0, b = 0, c = 60, d = 90)
- Normal blood pressure: function parameters: (a = 80, b = 90, c = 100, d = 120)
- Elevated blood pressure: function parameters: (a = 110, b = 120, c = 130, d = 140)
- High blood pressure: function parameters: (a = 130, b = 140, c = 200, d = 200)
- In this case, the membership functions will be trapezoidal, which allows for a plateau in the middle of each category, representing a gradual transition in blood pressure levels.

**Figure 5:**
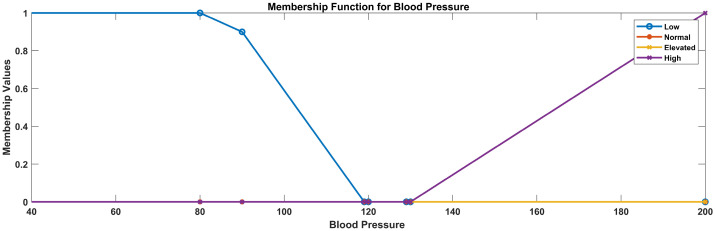
Membership functions for blood pressure.

In Figure 5, the blue line represents the membership values for low blood pressure, the purple line represents the membership values for high blood pressure, the yellow line represents the membership values for elevated blood pressure, and the red line represents the membership values for normal blood pressure.

b. Temperature

To define membership functions for temperature categories (low, normal, and high), triangular membership functions are employed given their suitability for this categorization. A triangular membership function is defined by three parameters: a (start), b (center), and c (end). The degree of membership rises linearly from ‘a’ to ‘b’ and then falls linearly from ‘b’ to ‘c’. The triangular membership functions defined for the specified temperature categories considering the data ranges were used after data analysis: 0–34, low; 35–38, normal; and 39, high.

- Low temperature: function parameters: a = 0, b = 0, c = 17
- Normal temperature: function parameters: a = 15, b = 20, c = 25
- High temperature: function parameters: a = 23, b = 30, c = 40

**Figure 6:**
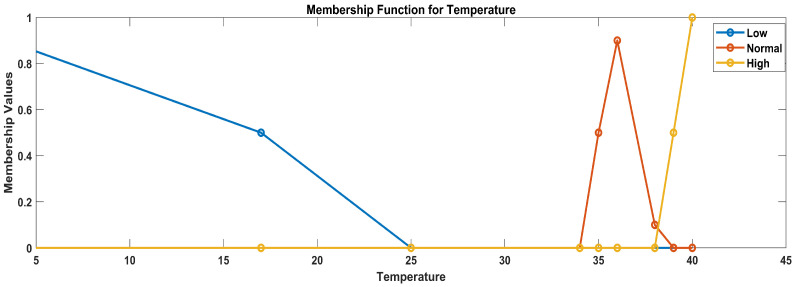
Membership functions for temperature.

In Figure 6, the blue line represents the membership values for low temperature, the blue line represents the membership values for high temperature, and the red line represents the membership values for medium temperature.

c. Glucose level

For defining the membership functions for glucose level categories (low, normal, elevated, and high), we use trapezoidal membership functions to represent the gradual transition between categories. The trapezoidal membership functions have four parameters: ‘a’, ‘b’, ‘c’, and ‘d’, where ‘a’ and ‘d’ are the start and end points of the plateau and ‘b’ and ‘c’ are the points where the membership function starts to rise and fall, respectively.

The trapezoidal membership functions for the specified glucose level categories of diabetic patients considering the data ranges mentioned below were used after data analysis: 0–69, low; 70–100, normal; 101–124, elevated; and 125, high.

- Low glucose level: function parameters: a = 0, b = 0, c = 40, d = 70
- Normal glucose level: function parameters: a = 50, b = 70, c = 90, d = 100
- Elevated glucose level: function parameters: a = 90, b = 100, c = 120, d = 125
- High glucose level: function parameters: a = 120, b = 125, c = 200, d = 200

**Figure 7:**
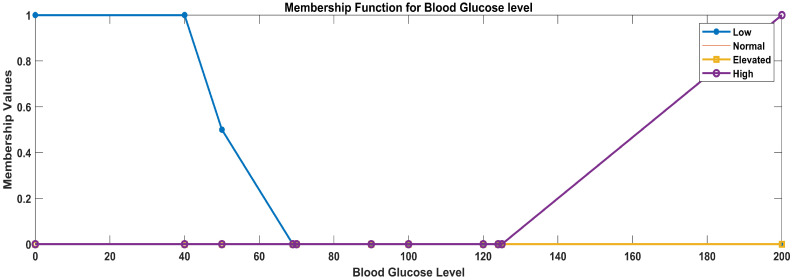
Membership functions for glucose level.

In Figure 7, the blue line represents the membership values for low blood glucose, the orange line represents the membership values for normal blood glucose, the yellow line represents the membership values for elevated blood glucose, and the purple line represents the membership values for high blood glucose.

d. Heart rate

For defining the membership functions for the heart rate categories (low, normal, and high), we used trapezoidal membership functions to represent the gradual transition between categories. The trapezoidal membership functions have four parameters: ‘a’, ‘b’, ‘c’, and ‘d’, where ‘a’ and ‘d’ are the start and end points of the plateau and ‘b’ and ‘c’ are the points where the membership function starts to rise and fall, respectively. The trapezoidal membership functions for the specified heart rate categories considering the data ranges were used after data analysis: 0–109, low; 110–155, normal; and 156, high.

- Low heart rate: trapezoidal membership function parameters: a = 0, b = 0, c = 90, d = 109
- Normal heart rate: trapezoidal membership function parameters: a = 100, b = 110, c = 145, d = 155
- High heart rate: trapezoidal membership function parameters: a = 150, b = 156, c = 200, d = 200

**Figure 8:**
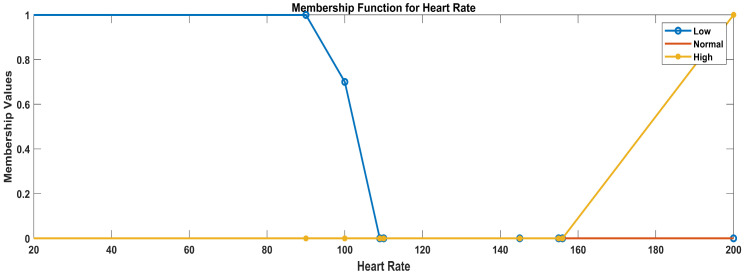
Membership functions for heart rate.

In Figure 8, the blue line represents the membership values for low heart rate, the red line represents the membership values for normal heart rate, and the yellow line represents the membership values for high heart rate.

e. Oxygen saturation

Let us define the trapezoidal membership functions for the specified oxygen saturation categories considering the data ranges that were used after data analysis: 0–89, critical; 90–93, worrisome; and 94, normal.

- Critical oxygen saturation: function parameters: a = 0, b = 0, c = 80, d = 89
- Worrisome oxygen saturation: function parameters: a = 85, b = 90, c = 95, d = 93
- Normal oxygen saturation: function parameters: a = 92, b = 94, c = 100, d = 100

**Figure 9:**
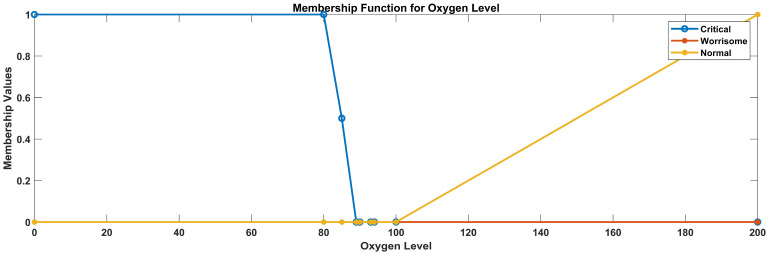
Membership functions for oxygen level.

In Figure 9, the yellow line represents the membership values for low oxygen level, the green line represents the membership values for normal oxygen level, and the red line represents the membership value for high oxygen level.

#### 3.3.2. Fuzzy Rule Base 

A set of rules were defined that connect the input fuzzy sets to the output fuzzy sets. The following rules typically use operators like AND, OR, and NOT to connect antecedents and consequents.

IF (Temp == Normal) AND (HR == Normal) AND (SpO_2_ == Critical) AND (BP == Elevated) AND (GL == Normal) THEN (Condition == Critical) (1)

IF (Temp == Low) AND (HR == Low) AND (SpO_2_ == Worrisome) AND (BP == Normal) AND (GL == Elevated) THEN (Condition == Unhealthy) (2)

IF (Temp == Normal) AND (HR == Low) AND (SpO_2_ == Normal) AND (BP == Low) AND (GL == Low) THEN (Condition == Very Critical) (3)

IF (Temp == Low) AND (HR == Low) AND (SpO_2_ == Normal) AND (BP == Low) AND (GL == High) THEN (Condition == Very Critical) (4)

IF (Temp == Low) AND (HR == Low) AND (SpO_2_ == Worrisome) AND (BP == Low) AND (GL == Normal) THEN (Condition == Very Unhealthy) (5)

IF (Temp == High) AND (HR == Normal) AND (SpO_2_ == Worrisome) AND (BP == Normal) AND (GL == Normal) THEN (Condition == Moderate Unhealthy) (6)

IF (Temp == Low) AND (HR == Normal) AND (SpO_2_ == Normal) AND (BP == Normal) AND (GL == Normal) THEN (Condition == Unhealthy) (7)

#### 3.3.3. Inference Engine

The fuzzy rules were defined based on doctors’ knowledge and data analysis. These rules are used to infer new fuzzy sets based on the fuzzy values obtained during fuzzification. Membership functions in fuzzy logic define how each data point from the universe of discourse (the range of possible values) is associated with a degree of membership to a particular fuzzy set. According to this context of sensor data, membership functions help in fuzzifying the crisp sensor readings into fuzzy values.

#### 3.3.4. Rule Evaluation

The degree of membership for each rule based on the sensor data’s fuzzified values is evaluated. There are many other rules based on combinations of factors affecting health with different membership values.

#### 3.3.5. Defuzzification

The fuzzy output set is converted into a crisp output, as shown in Table 5.

#### 3.3.6. Output

According to all the inputs and after considering them aggregately, the predicted crisp value of the condition is obtained, as shown in Table 5. According to the condition status, the doctor can take further clinical action, make recommendations and dosage changes, especially for diabetic patients whose medication is dependent on current blood sugar values, and modify existing medication.

### 3.4. Implementation Details 

The circuit diagram below includes the initial sensing unit, where the received analog signal undergoes filtering. This signal is received from a photodetector containing approximately seven lights. The electric current from the photodetector is converted to voltage, in which process the resistances of different loads are used. The low-pass filter allows low-frequency components to pass through while attenuating high-frequency noise. The cut-off frequency of the LPF determines the point at which the filter starts dealing with higher-frequency components. The cut-off frequencies for the LPF in NIR glucose sensing range from 2 Hz to 5 Hz, but the exact value depends on patient temporal changes and learning of patient patterns over time. The high-pass filter, on the other hand, permits high-frequency components to pass while attenuating low-frequency noise. The HPF cut-off frequencies range from 0.01 Hz to 0.1 Hz, or are even lower, depending on the characteristics of the noise sources, according to which the desired frequency range is adjusted for glucose measurement.

Impact on signal quality: The LPF effectively reduces noise and unwanted low-frequency components that may arise from various sources, such as motion or baseline drift. It ensures that the signal primarily contains the relevant spectral information associated with glucose. Baseline drift can be caused by variations in instrument conditions, temperature, or skin tissue properties. The LPF helps remove this drift, allowing for more accurate glucose measurements. The HPF helps attenuate high-frequency noise, such as electrical interference or measurement artifacts. By eliminating high-frequency noise, the HPF contributes to improved signal quality. The frequency from the high-pass and low-pass filters is adjusted so that the minimum voltage is gained and the correct value is calculated. After this, an amplifier is attached with the Arduino Nano that converts the analogue values to digital values, which are then translated into textual actual values of the sensor.

The experimental setup and protocols: Certainly, providing details on the experimental setup and protocols used for the validation of the proposed healthcare system with standard apparatus data is crucial for understanding the evaluation process. Below is a dedicated subsection that outlines the experimental setup and protocols and includes a comparison of tables for clarity Table 6.

**Figure 10:**
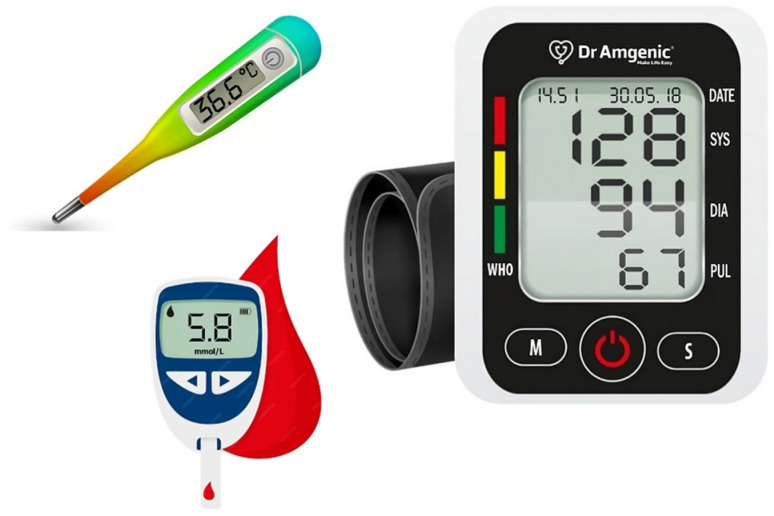
Standard clinical apparatus.

For validation of the proposed smart sensing healthcare system, standard apparatus data were collected using reputed medical equipment. The measuring parameters involved were blood glucose level, oxygen level, blood pressure, temperature, and heart rate. Standard clinically validated apparatus were used for the standard data collection [40], as shown in Figure 10. In Figure 10, 1 represents the blood pressure measuring apparatus, 2 represents the glucometer, and 3 represents the digital thermometer. Sample population data were collected from a coherent group considered to be representative of the target population. The parameters for determining a similar sample population were age, gender, and health condition (diabetes).

**Figure 11:**
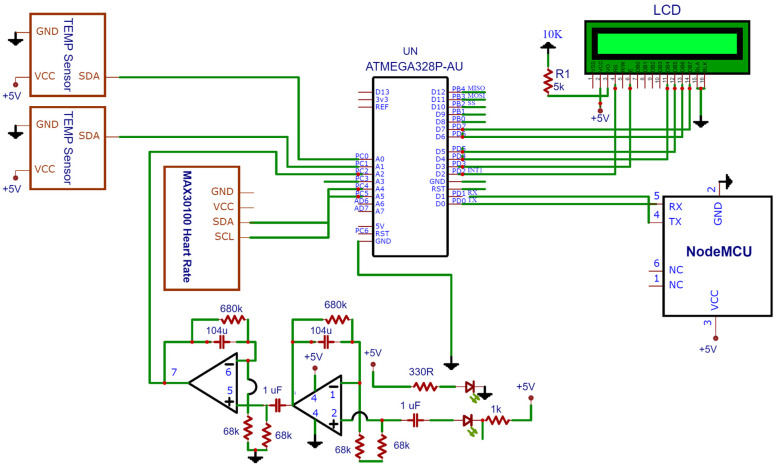
Connected IoT circuit diagram.

Figure 11, a connected IoT circuit diagram, illustrates the communication of all the sensors and the controller to the LED in the proposed architecture. The subsequent block diagram in the same figure depicts the entire flow of data and procedures within the architecture.

#### Glucose Measuring Sensor for Diabetic Patients

The non-invasive method for glucose measurement minimizes costs related to voltage, frequency (in Hz), and resistance. It achieves this while effectively managing noise and utilizing an amplifier to enhance the output signal. The NIR sensor is a non-invasive sensor that measures the interaction of near-infrared light with the glucose molecules in the body. The device emits light onto the skin and a detector measures the amount of light absorbed by glucose using an NIR light source with a 940 nm wavelength. Glucose levels are calculated by measuring the light signal intensity after penetration of the finger. The Beer–Lambert law serves as the foundational principle for calculating the anticipated measurement of glucose levels in the blood. The light that penetrates finger tissue obviously scatters after penetration and passes through blood. By measuring the absorbance of light and the concentration of blood fluid and the length of the path travelled by light, the blood glucose level is determined. We also validated the values of glucose in blood with standard invasive glucometer measurements [41,42].

Hardware: NIR glucometers consist of a light source, a detector (a photodetector), and the necessary optics to direct light onto the skin and collect the reflected or transmitted light. Factors that affect NIR sensors are wavelength, light penetration, effects on biological tissues, skin thickness, and light intensity. In the current work, all these factors are considered ideally. To address these challenges, several specific measures and techniques can be employed, like calibration; data processing; spectral retreatment; model selection and optimization; continuous glucose monitoring; hydration compensation; multi-spectral approaches; controlling environmental factors like temperature, humidity, and ambient light during measurements to reduce interference from external sources; user education; and clinical validation. 

Applying a calibration method to blood glucose data using an ML model involves the use of supervised learning techniques. In this case, supervised learning aims to build a predictive model that needs to be mapped with numerical measurements (features) to actual blood glucose values (targets). A data set is gathered that includes two key components: numerical measurements (features) and actual blood glucose values (targets). The features come from a device like an NIR sensor, and the targets are obtained through traditional blood glucose testing methods. The data are preprocessed to ensure that they are suitable for ML. This may involve handling missing values, scaling the features, and addressing outliers. The data set is then divided into training and validation sets. The training set is used to train the ML model; the validation procedure is used for benchmarking of the proposed system to evaluate the final model’s performance. ML algorithms are applied and trained using the training data set and validated with the standard data obtained via the standard clinical procedure. To reduce the impact of these factors, we performed 3 steps to overcome the challenges.

- Adjusted the wavelength to the optimal range: 700 nm to 2500 nm;
- Compared the data with standard and actual readings for validation;
- User education.

Challenges that need to be addressed in designing and implementing the proposed system are:

Managing patient acceptance and collaboration in the practical implementation of a non-invasive glucose measurement system using NIR light in clinical settings is necessary for the system’s success, as is patient education regarding how the system works, as well as its positives and negatives, and ensuring the proper placement of sensors. For patient convenience, a non-invasive and painless method is used. Privacy and confidentiality concerns need to be addressed. Feedback should be sought if patients face any issues or discomfort. System readings are visible to the patient on screen to motivate the patient to perform the correct cooperative behavior. All readings on screen appear in a few milliseconds, creating curiosity and interest in using the system.

### 3.5. Dataset Details

Data from diabetic patients are collected using the proposed system by patients placing their hands on each sensor. Within seconds, data from all the sensors are gathered, displayed on the LED, and uploaded to the server. The data uploaded to the server are also viewable by practitioners, who can suggest taking medicine accordingly. Data were also collected via standard apparatus, like blood pressure apparatus, thermometers, oximeters, ECG machines, and blood sampling for diabetic sugar-level reports that may be received after a few hours or on another day. The ranges and labels of data are shown in Table 7. There are challenges in long-term continuous monitoring and patient self-maintenance.

The proposed system design is user-friendly and comfortable to use. Patients just need to wear the sensor on the finger or thumb, wherever is comfortable, for blood glucose measurement. For other measures, patients just need to put their fingers on the sensors. For correct sensor placement, the patient can attach the sensors with the hand or can wear gloves. In case the patient wants to wash their hands or cannot wear the sensor on the hand, they can also wear it on one of their toes. 

Patients can view all readings on the screen attached to the system, which can help patients to take proper doses and be aware of their condition and control their diet accordingly.

User education and training also help to mitigate the issues that arise in long-term use. The guidelines for using the sensor system are: try not to bring the sensors into contact with water while wearing them and keep the batteries adequately charged or have a power supply nearby to ensure continuous monitoring.

The data collected (see Table 5) through the designed system were first classified and annotated with different labels that would be helpful for data training, and some ML techniques were implemented that predict the diabetic patient’s condition as critical, unhealthy, very critical, very unhealthy, or moderate(Table 7). Data training, manipulation for missing values, classification of data, and implementation of some ML algorithms were performed in Jupiter notebook.

Creating a full-fledged priority queue implementation in the Arduino Nano can be complex due to memory constraints and the lack of direct support for advanced data structures. However, we can simulate a simplified version of a priority queue to handle the given scenario. After collecting data, the second step is to apply the smart priority algorithm to this sensed data, as shown in Algorithm 1 This smart algorithm is also a novel feature of our work designed to process and effectively utilize a large amount of data. The algorithm used is described in Priority Algorithm 1 on sensed data.
**Algorithm 1** To filter and prioritize sensor data over the network**1**   **Input: 1.** Initialize constants and data structures:          Set *Normal_Sensor1_Min = Vmin*          Set Normal*_ Sensor1_Max = Vmax*          Set Normal*_Sensor2_Min = Vmin*          Set Normal*_ Sensor2_Max = Vmax (for all sensors)* **2**   **Input 2:** Initialize sensor data array:
      Initialize an array sensor queue of size 5 to hold sensor data objects for each sensor
**3**   **Loop:**     Read data from each sensor and update sensor queue      **For each sensor:**       Read sensor data         Update sensor queue with the new data (sensor ID, value, priority) **4**   **Check for out-of-range sensor data:**       **If sensor data are out of range:**         Set higher priority for the data packet          Update sensor queue priority for the respective sensor **5**   **Process the priority queue:**       **For each sensor in sensor queue:**         **If priority is high:**           Trigger alert and send data packet          Alerting mechanism (sound)          Send the data packet with higher priority **6**   **Repeat the loop** (periodically to continuously monitor the sensors and handle alerts)       End algorithm

Priority Algorithm 1 arrays are used to represent sensor data along with a basic mechanism to prioritize alerts for out-of-range sensor readings.These readings are then calibrated by fuzzy logic that impacts the health condition outcome.as shown in Table 8.

## 4. Results

The results section corresponds to the account of the methodology given in Section 3. According to the methodology section organization, firstly, the system validation process is presented in Figure 3. So, the first section on results comprises the system validation with standard system analysis and evaluation measures. Secondly, for the smart decision support system, the hybrid AI and ML approach is discussed along with the layered architecture and fuzzy logic-based decision support. Thus, in the second section on results, fuzzy logic-based linguistic variables and class comparison results are presented which also give an in-depth comparison between the standard system and the proposed system (see Table 8). Thirdly, in Section 3.4, the system’s implementation details are discussed regarding all sensors and the microcontroller connectivity. The results for the blood glucose sensor and priority algorithm are also discussed in this section. Fourthly, data set details are discussed, and the data set analysis and histograms are also presented. At the end, an overall impact analysis along with a few results are discussed that give insightful knowledge to experts and practitioners regarding health condition decision making.

### 4.1. System Validation 

Sample population data were collected from a coherent group of considered to be representative of the target population. The parameters for the sample population were age, gender, and health condition (diabetes). Considering the same parameters for both measurements, both system measurements were compared with respect to evaluation criteria, including precision, accuracy, F1 score, and recall. Further comparative analysis is also given in the following table on the basis of a statistical analysis.

In the comparison (Table 9), the means and standard deviations of measurements obtained from both the standard apparatus and the proposed healthcare system are presented for each health parameter. The mean absolute error (MAE) is provided as a measure of the absolute difference between the two sets of measurements. Smaller MAE values indicate better agreement between the proposed system and standard apparatus.

System validation or benchmarking was performed by comparing the data collected with the proposed system with those collected with the standard apparatus and by applying the proposed methodology to both sets of data, as shown in Figure 3.

In Table 10, the accuracy, precision, F1, and recall scores calculated for the proposed portable IoT system for diabetic patients are presented. The support vector algorithm achieved scores of 70% for accuracy, 30% for F1 score, 27% for precision, and 36% for recall. Gaussian Naïve Bayes achieved 55% for accuracy, 38% for F1 score, 37% for precision, and 48% for recall. The decision tree classifier achieved 80% for accuracy, 53% for F1 score, 50% for precision, and 60% for recall.

Correspondingly, the random forest classifier achieved 85% accuracy, which is the highest level in comparison with the other ML algorithms. The Bernoulli Naïve Bayes algorithm achieved 55% for accuracy, 20% for F1 score, 31% for precision, and 80% for recall, like the results presented in [8,29].

**Figure 12:**
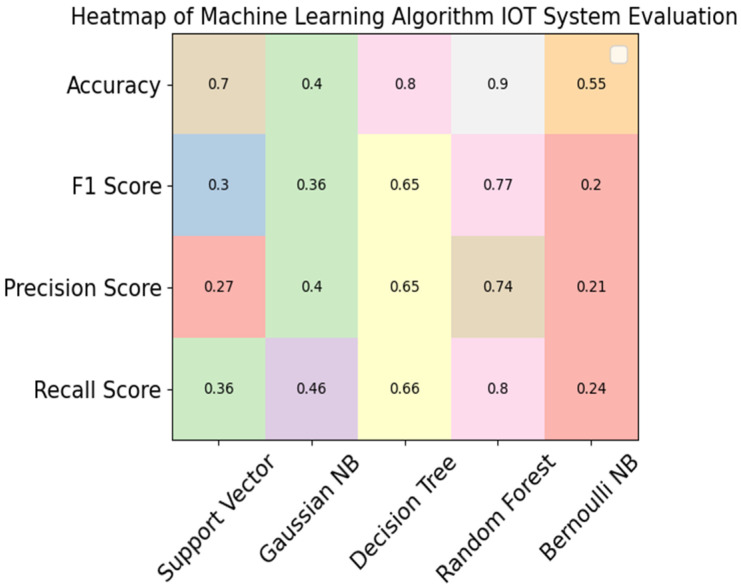
Comparison of ML algorithms for IoT proposed system.

Figure 12 presents a comparison of the support vector algorithm, Gaussian NB, the decision tree classifier, the random forest classifier, and Bernoulli NB in terms of accuracy, F1, precision, and recall scores for the proposed system.

**Figure 13:**
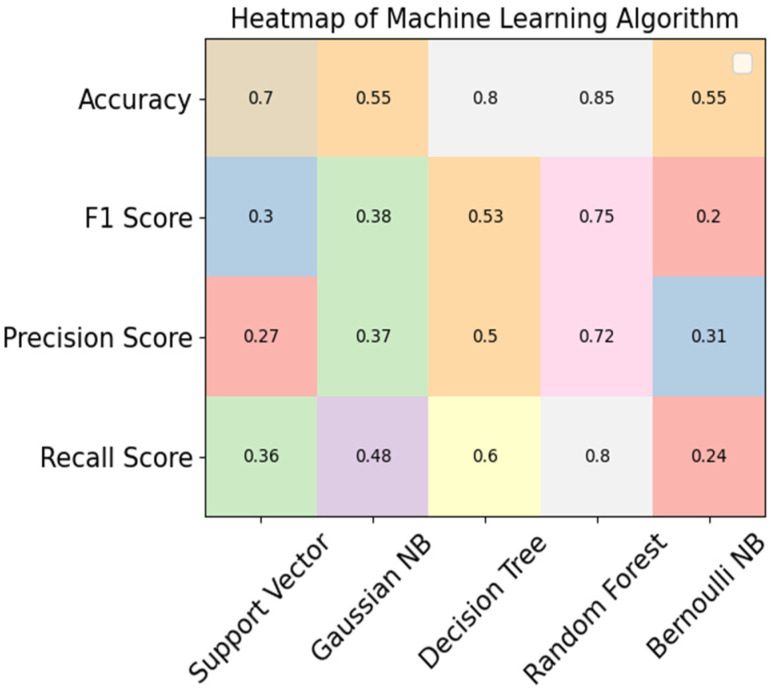
Heatmap comparison of ML algorithms for standard data.

In Table 11, accuracy, precision, F1, and recall scores calculated for the proposed portable IoT system are presented. The support vector algorithm achieved 70% for accuracy, 30% for F1 score, 27% for precision, and 36% for recall Gaussian Naïve Bayes achieved 40% for accuracy, 36% for F1 score, 37% for precesion, and 48% for recall. The decision tree classifier achieved 80% for accuracy, 53% for F1 score, 50% for precision, and 60% for recall. Correspondingly, the random forest classifier achieved 90% for accuracy, which was the highest level in comparison with the other ML algorithms. The Bernoulli Naïve Bayes algorithm achieved 55% for accuracy, 20% for F1 score, 21% for precision, and 24% for recall. The data shown in Table 11 are also illustrated in Figure 13.

**Figure 14:**
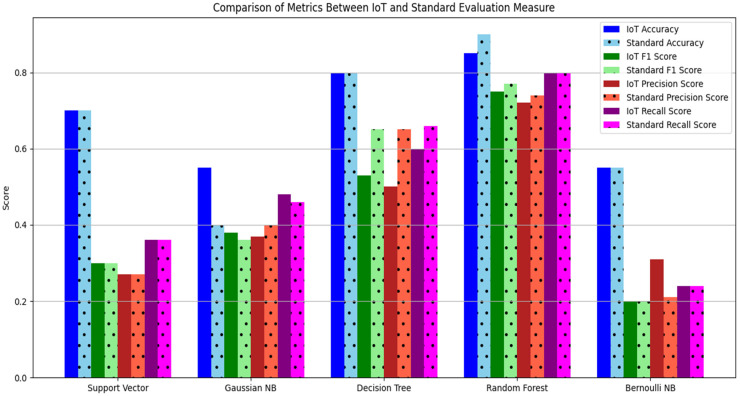
Comparison of ML algorithms.

Figure 14 represents a comparison of the support vector algorithm, Gaussian NB, the decision tree classifier, the random forest classifier, and Bernoulli NB in terms of accuracy, F1, recall, and precision scores for both sets of data collected with the proposed system and the standard apparatus for diabetic patients. Figure 14 represents a comparison of the ML algorithm results for both the standard and proposed system approaches in terms of accuracy, precision, F1, and recall scores.

**Figure 15:**
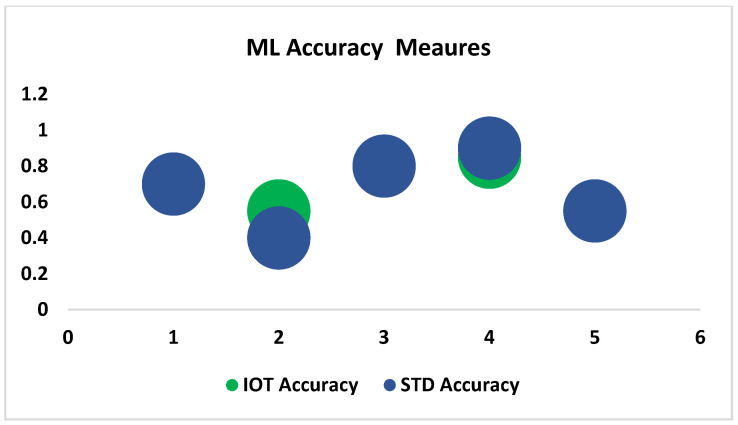
Comparison of accuracy scores of the proposed and standard systems with respect to ML data.

In Table 12 and Figure 15, the accuracy scores for the proposed system achieved by all five ML algorithms are compared with the standard apparatus measurements. According to the table, the support vector algorithm, the decision tree classifier, and Bernoulli Naïve Bayes gave similar results, which demonstrates the validity of the proposed system.

### 4.2. Results Analysis for the ML and AI Layers

**Figure 16:**
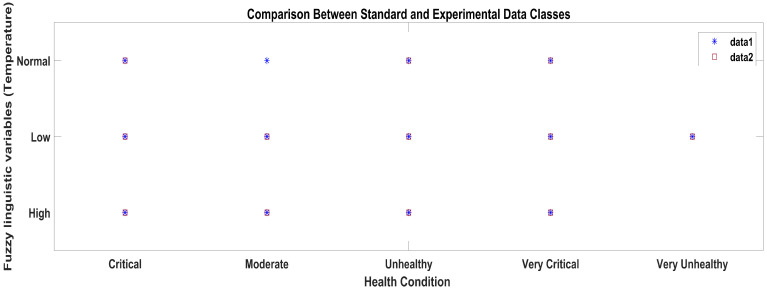
ML classification comparison between standard and proposed systems.

According to the methodology, the results obtained from the AI layer and the ML layer are discussed in this section. The output crisp values obtained from the AI layer serve as inputs to the ML layer of the layered architecture, which predicts the health condition considering all the vital signs data received from the AI layer, as shown in Figure 16. In Figure 16, the star symbols represent the data obtained with the standard apparatus and the red squares represent the data obtained with the proposed system. 

**Figure 17:**
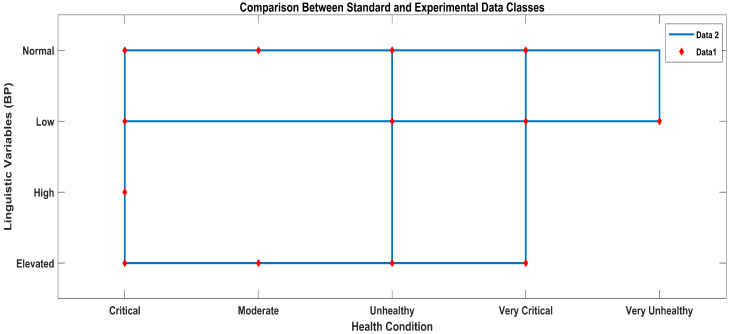
ML classification comparison between standard and proposed systems.

In Figure 17, a staircase line plot and a scatter plot give separate visualizations of the different data sets and also the relationship between the two data sets (i-e) for the standard and proposed systems. The red scatter points give the correlations between the linguistic variables and the ML classes of the standard blood pressure data, and the blue stairs show the correlations between the linguistic variables of the data labels and the ML classes of the proposed experimental blood pressure data. 

**Figure 18:**
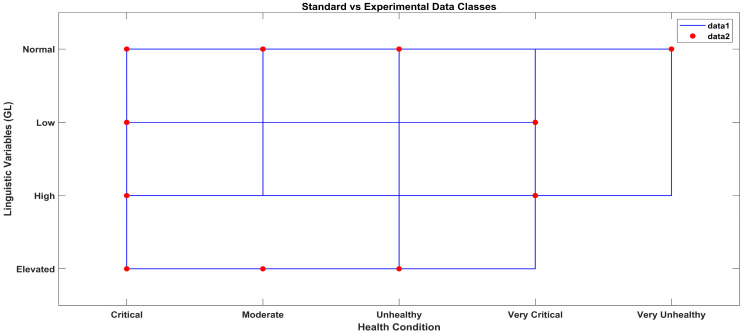
ML classification comparison between standard and proposed systems.

Figure 18 shows a staircase line plot and a scatter plot giving separate visualizations of the different data sets and also the relationship between the two data sets (i-e) collected with the standard and proposed systems. The red scatter points give correlations between the linguistic variables and the ML classes of the standard blood glucose data, and the blue stairs show the correlations between the linguistic variables of the data labels and the ML classes of the experimental blood glucose data obtained with the proposed system. 

**Figure 19:**
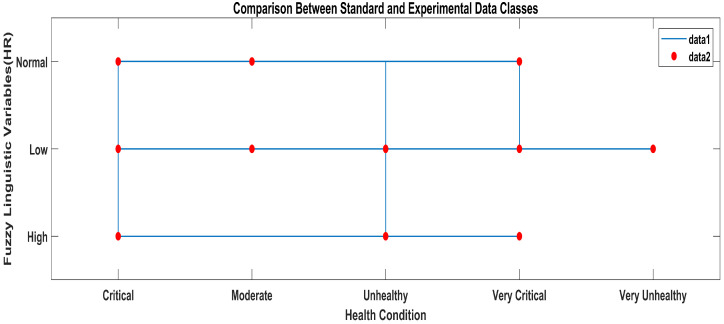
ML classification comparison between standard and proposed systems.

If one standard data set represents continuous variables and the other represents categorical variables, their combination can highlight variations in both sets of data simultaneously. Figure 19 shows a staircase line plot and scatter plot giving separate visualizations of the different data sets and also the relationship between the two data sets (i-e) collected with the standard and proposed systems. The red scatter points give correlations between the linguistic variables and ML classes of the standard heart rate data, and the blue stairs show correlations between the linguistic variables of the data labels and ML classes of the experimental heart rate data obtained with the proposed system. 

**Figure 20:**
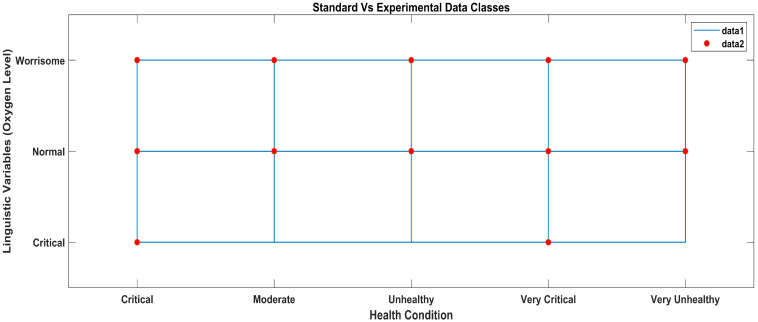
ML classification comparison between standard and proposed systems.

Figure 20 shows a staircase line plot and a scatter plot giving separate visualizations of the different data sets and also the relationship between the two data sets (i-e) collected with the standard and proposed systems. The red scatter points give correlations between linguistic variables and ML classes of the oxygen level data obtained with the standard apparatus, and the blue stairs show correlations between the linguistic variables of the data labels and ML classes of the experimental oxygen level data obtained with the proposed system. 

### 4.3. Results for the Implementation Details and Data Analysis

**Figure 21:**
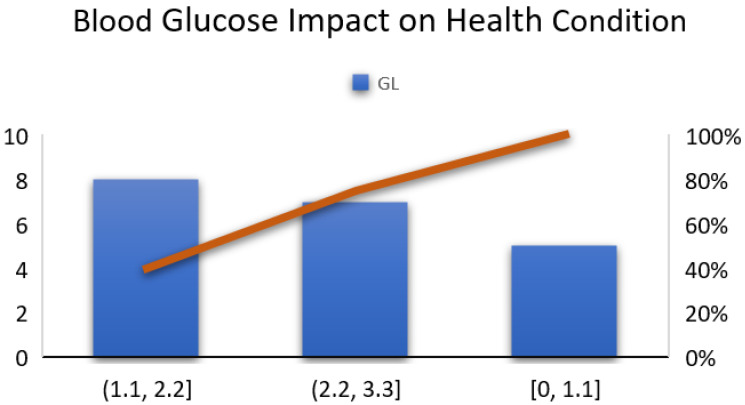
Impact of blood glucose level on health condition.

According to the description of the methodology on page 17, the implementation details of the hardware system and the experimental setup are described in this section. A blood glucose non-invasive NIR sensor was designed with low voltage and an adjusted wavelength. The output results of this sensor and its impact on overall decision making and health prediction are shown in Figure 21. 

According to our layered methodology, we collected data through our designed hardware system, then developed a software layer to organize the data and process them through ML and AI for smart decision making. Thus, histograms were plotted for the proposed model data, which were validated through the standard apparatus to make them clearer and more insightful. Histograms for these health parameters can be valuable tools for healthcare professionals to understand the distribution of values within a population or track changes in individual patients over time. They can aid in identifying potential health issues, assessing treatment effectiveness, and making informed decisions about patient care.

**Figure 22:**
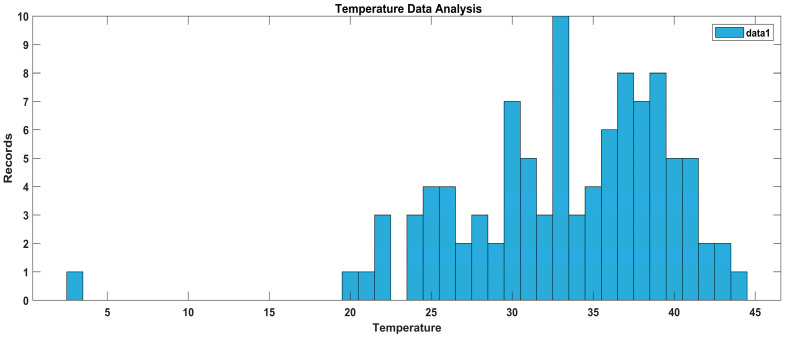
Data analysis of temperature.

In Figure 22, a data distribution is presented, by means of which we can conclude that the data on temperature for diabetic patients in the experimental setup are clustered between 20F and 43F. There are a few readings of outliers and exceptional cases which we handle in the data preprocessing phase for machine learning. 

**Figure 23:**
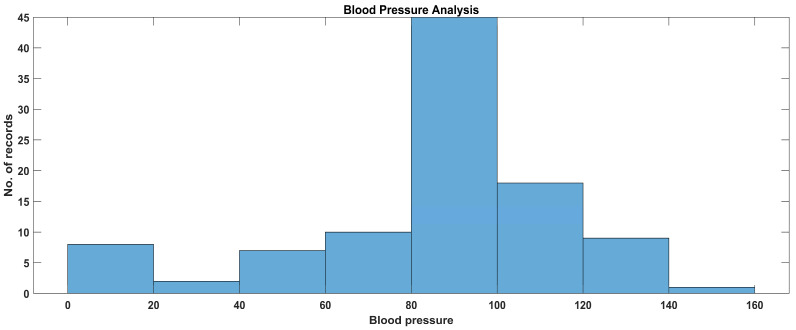
Data analysis of blood pressure.

Similarly, in Figure 23, the data disparity and distribution are shown, and the distribution is for realistic data; thus, most of the data are clustered in the 40 mmHg to 160 mmHg value range, and according to the graph there are some emergency cases regarding blood pressure that need to be handled, but on average a normal blood pressure range between 80 mmHg and 120 mmHg existed in the sample experimental population. 

**Figure 24:**
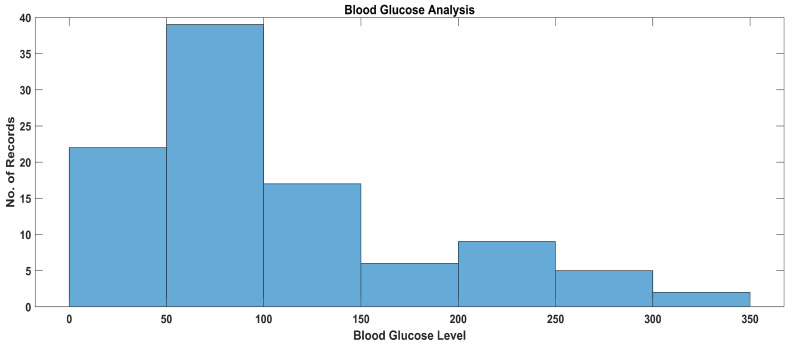
Data analysis of blood glucose levels.

In Figure 24, the data distribution of blood glucose levels in diabetic patients is presented. This distribution graph gives a clear picture of recent records considering patients for practitioners, who can suggest further diagnosis and medications accordingly.

**Figure 25:**
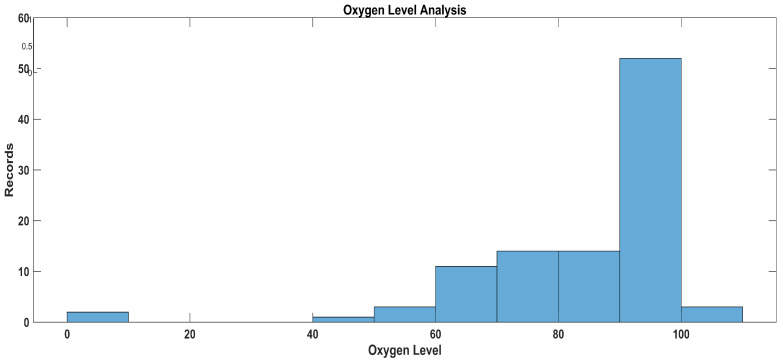
Data analysis of oxygen levels.

In Figure 25, the data disparity and distribution are shown, and this distribution is for realistic data; thus, most of the data are clustered in the 60% to 100% mmHg value range.

**Figure 26:**
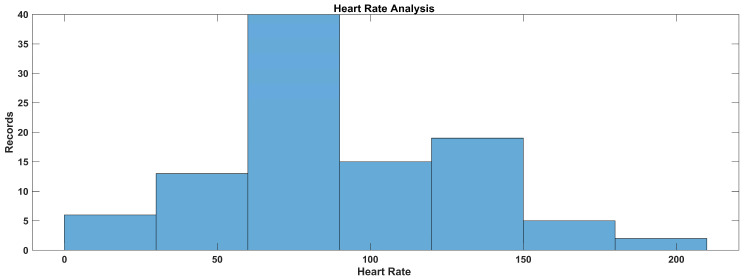
Data analysis of heart rates.

In Figure 26, the heart rate value distribution is presented. According to the figure, the data are normally distributed between 50 BPM and 220 BPM.

**Figure 27:**
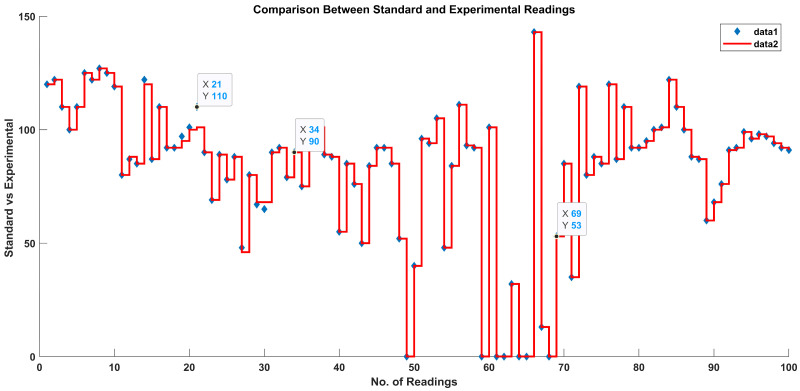
Impact analysis of vital signals on predicted health condition for a large data set.

Now, some results are shown below for an impact analysis of the data at different layers. In Figure 27, the overall impacts of blood glucose vital signals on predicted health conditions for the standard apparatus data and for the proposed-system-generated blood glucose data are compared. To validate the specially designed NIR sensor, comparative results are shown in Figure 27, in which the blood glucose measurements are compared. The blood glucose measurements obtained by the NIR sensor are in mmol/L unit, and the clinical apparatus takes measures in mg/dL. Thus, we needed to convert our values into mg/dL for further processing. The X-axis represents the number of patients involved. This graph presents 25 readings for comparison. A few ID values vary in comparison with the other IDs. It is true that the readings obtained via the standard and invasive method are more accurate, but we designed the NIR-based sensor to avoid the inconvenience of needle penetration for blood sampling for the measurement of blood glucose levels. The Y-axis represents the blood glucose readings. The green lines represent the readings of the proposed sensor, and the orange lines represent the readings obtained via the standard method.

**Figure 28:**
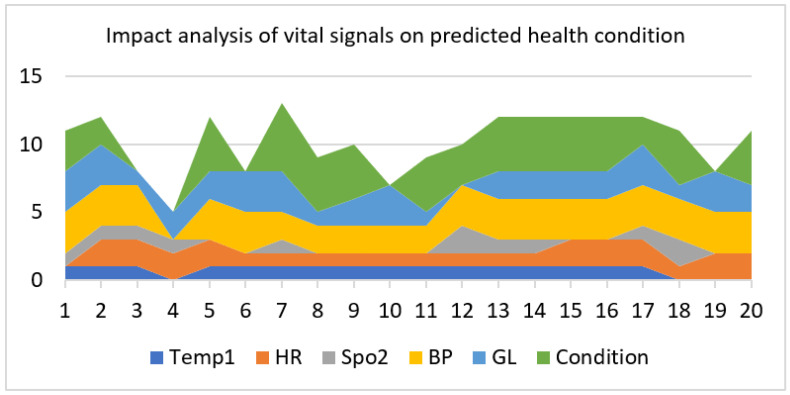
Impact analysis of vital signals on health decision.

Figure 28 gives an in-depth pictorial impact analysis of all vital-sign annotated data ranges on annotated health prediction through the ML algorithms collectively. The stacked layered area plot shows each signs’ contribution and impact through the area covered in width and in length along the X-axis. In Figure 28, the dark blue color represents temperature annotated data ranges from 0 to 2, the orange color represents heart rate, the grey color represents oxygen level, the yellow color represents blood pressure, the light blue color represents blood glucose level, and the grassy green color represents predicted health condition through the ML algorithms.

**Figure 29:**
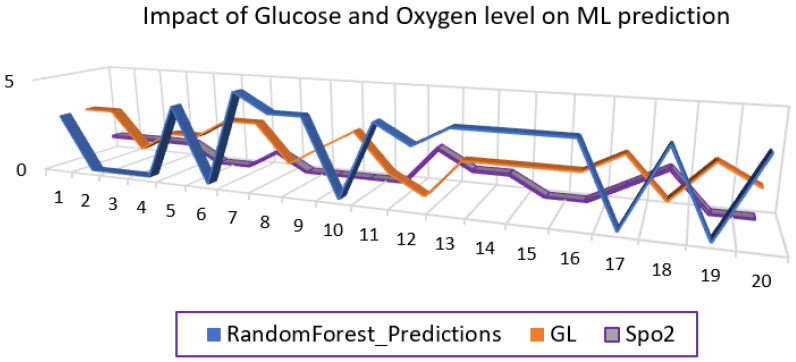
Impact of glucose and oxygen levels on ML prediction.

In Figure 29, the annotated range classes of vital health signs that have the greatest effect on the diabetic patient’s health condition are taken for analysis for each ML algorithm. One set of the random forest algorithm’s prediction results are plotted against the considered vital signs. Such results and graphs can only be available to the practitioner and the patient on request and according to the prescribed condition. The blue color represents the random forest prediction algorithm output, the orange color represents blood glucose level, and the purple color represents the oxygen level in the blood.

**Figure 30:**
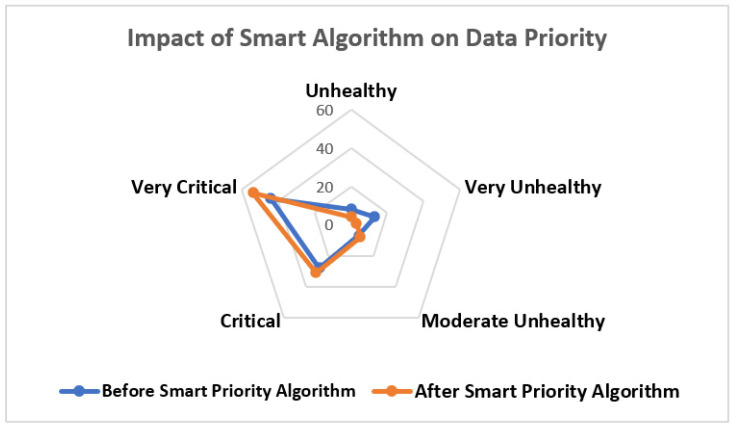
Impact analysis of smart priority algorithm.

Figure 30 shows the impact analysis of the smart priority algorithm evaluated on sensed data. The analysis was performed on the first 100 ID records of diabetic patients. The initial 100 records may represent a pilot or preliminary study to evaluate the algorithm’s performance. This allows for an initial assessment of the algorithm’s effectiveness and impact. Initiating with a smaller data set could be a prudent approach to fine-tune the algorithm and identify potential issues or challenges before scaling up to a larger data set. Each corner of the radar represents the health conditions predicted, and each iterated cirrcle represents the no. of records of diabetic patients. The blue line sections represent data received before applying the priority algorithm, and the orange line sections represent the impacts after applying the priority algorithm. After applying the algorithm, the critical and very critical increasing from unhealthy to critical states of predicted health conditions are compared to the numbers without applying the smart algorithm. Thus, the results show that the in-emergency situation values get priority as compared to the normal health measurements. The novel and significant proposal that has been presented here has not been applied up till now in healthcare systems. The first 100 ID records were taken to analyze the clear impact of the priority algorithm and to obtain presentable results. It can be applied to all data, but it is only to be applied according to doctor’s preferences for diabetic patients.

## 5. Conclusions

We conclude our paper by saying that a smart healthcare monitoring system is an essential need, and with the passage of time the number of patients suffering from diseases like diabetes and COVID-19 is increasing. To control their condition and for safety measures, diabetic patients must have a portable system that can be reviewed by doctors repeatedly. The proposed system covers five essential factors of diseases, namely, blood glucose level, blood pressure, heart rate, oxygen level in the blood, and temperature. The blood glucose sensor for diabetic patients is non-invasive, frequent measurements using an invasive method not being possible. We validated our system with standard procedure apparatus/machine data and applied ML algorithms for the prediction of the health condition of patients. Comparisons in terms of accuracy, F1 score, recall, and precision were performed, which showed that the proposed system gave 98 percent similarity to the results obtained via standard apparatus and machines. Thus, the proposed system predicts diabetic patients’ health conditions as critical, very critical, unhealthy, very unhealthy, or moderately unhealthy. In future work, telehealth enhancement factors can be introduced. ML and AI are being used already. Deep learning and another prediction algorithm, OIESGP, exist in healthcare, but not for diabetic patients. We will try to achieve implementation by adapting new and innovative algorithms. New innovative sensors can also be introduced with evolving technologies which are helpful in performing visible camera monitoring for chronic and emergency patients, making telehealth more accurate and effective.

## Figures and Tables

**Table 1:** Comparison between smart health and simple health [3,4,9,10].

Smart Health Application Pillars	Simple Health Applications Pillars
Patient-centered care	Basic health information
Interoperability	Health and fitness tracking
Data integration	Medication reminders
Telehealth and telemedicine	Simplified user interface
Wearable and sensor technologies	Health records
Data analytics and machine learning	Accessibility and inclusivity
Telemonitoring	Privacy and data protection
Health information exchange	-

**Table 2:** Advanced feature analysis between smart and simple health applications [11,12,13,14,15].

	Smart Health Applications	Simple Health Applications
Complexity	Smart health applications are more complex and comprehensive, covering a wide range of healthcare aspects.	Simple health applications are more focused and user-friendly.
Data integration	Smart health apps emphasize the integration of various healthcare data sources.	Simple health apps typically handle user-generated data only.
Advanced technologies	Smart health applications incorporate advanced technologies like AI, ML, and sensor data.	These technologies are less common in simple health applications.
Remote care	Remote monitoring, telehealth, and telemedicine are core components of smart health applications, enabling more advanced care delivery.	These systems enable less advanced care delivery.
Target audience	Smart health applications may target healthcare professionals and patients with specific health needs.	Simple health applications are designed for a broader consumer audience.

**Table 4:** Articles on HCDs and comparison with proposed work.

Feature	Comparative	Study Analysis		Proposed Work
Focus of study	Remote patient monitoring and diagnostics [22,26]	Chronic disease management [20]	Telemedicine and consultation [32]	Smart sensing and remote monitoring of diabetic patients
ML algorithms used	SVM, random forest, KNN	Deep learning (CNN, LSTM)	Decision trees, Naïve Bayes	Support vector algorithm, Gaussian NB, decision tree, random forest, Bernoulli NB
Data collection approach	Wearable devices, health sensors	IoT sensors, electronic health records	Remote monitoring devices, health wearables	Wearable health sensors (5 sensors, including a specially designed glucose sensor for diabetic patients)
Personalization level	High	Medium	High	High
Real-time alerts	Yes	Yes	Yes	Yes
Patient engagement strategy	Personalized recommendations, gamification	Health tracking, goal setting	Educational content, interactive interfaces	Beep alerts, graph readings are available
Scalability and accessibility	Scalable, cloud-based	Scalable, no cloud access	Scalable	Scalable, cloud access
Decision support system	No	No	No	Yes
Smart data processing	No	No	No	Yes (give priority to emergency data)

**Table 5:** Conversion of fuzzy output to crisp output.

ID	Temperature	Heart Rate	Oxygen Level	Blood Pressure	Glucose	Crisp Value of Condition
1	Normal	Normal	Critical	Elevated	Normal	0
2	Low	Normal	Worrisome	Elevated	Low	1
3	Low	Low	Worrisome	Normal	Elevated	3
4	Low	Normal	Worrisome	Normal	Low	0
5	Low	Low	Normal	Normal	Elevated	3

**Table 6:** User data set and demographics.

Size	Age	Gender	Ethnicity	Medical Condition	Physiological Data
150	22–60	45% female, 55% male	Punjab, Pakistan	Diabetes	Heart rate, oxygen level, blood glucose level, temperature, and blood pressure

**Table 7:** Ranges and labels for classification.

Sensor	Ranges	Label
Temperature	0–34	Low
Temperature	35–38	Normal
Temperature	39 and above	High
Blood pressure	0–90	Low
Blood pressure	91–119	Normal
Blood pressure	120–129	Elevated
Blood pressure	130 and above	High
Heart rate	0–109	Low
Heart rate	110–155	Normal
Heart rate	156 and above	High
Glucose level	0–69	Low
Glucose level	70–100	Normal
Glucose level	101–124	Elevated
Glucose level	125 and above	High
Oxygen saturation	0–89	Critical
Oxygen saturation	90–93	Worrisome
Oxygen saturation	94	Normal

**Table 8:** Calibration of sensor data with fuzzy logic system.

Temperature	Heart Rate	Oxygen Level	Blood Pressure	Blood Glucose Level	Condition
Normal	Normal	Critical	Elevated	Normal	Critical
Low	Normal	Worrisome	Elevated	Normal	Critical
Low	Low	Worrisome	Normal	Normal	Unhealthy
Normal	Low	Normal	Low	High	Very critical
Low	Low	Critical	Normal	Low	Very critical
Low	Low	Worrisome	Low	Normal	Very unhealthy
High	Normal	Worrisome	Normal	Normal	Moderate

**Table 9:** Data set validation.

Parameters	Standard Apparatus Measurement (Mean ± SD)	Proposed Apparatus Measurement (Mean ± SD)	MAE (Mean Absolute Error)
Blood glucose level	(104 ± 74.58) mg/d	(99.72 ± 78) mg/d	3.85 mg/dL
Blood pressure	(83.47 ± 31.69) mmHg	(83.36 ± 31.59) mmHg	0.21 mmHg
Heart rate	(87.12 ± 40.2) bpm	(86.73 ± 39.75) bpm	0.84 bpm
Oxygen level	(83.96 ± 18.24) %	(84.17 ± 18.36) %	0.33%
Temperature	(33.24 ± 6.55) F	(33.28 ± 5.92) F	0.67 F

**Table 10:** Evaluation measures for the proposed IoT system.

ML Algorithms	Accuracy	F1 Score	Precision	Recall
Support Vector Algorithm	0.70	0.30	0.27	0.36
Gaussian NB	0.55	0.38	0.37	0.48
Decision Tree	0.80	0.53	0.50	0.60
Random Forest	0.85	0.75	0.72	0.80
Bernoulli NB	0.55	0.20	0.31	0.24

**Table 11:** Evaluation measures for standard apparatus data.

ML Algorithms	Accuracy	F1	Precision	Recall
Support Vector Algorithm	0.70	0.30	0.27	0.36
Gaussian NB	0.40	0.36	0.40	0.46
Decision Tree	0.80	0.65	0.65	0.66
Random Forest	0.90	0.77	0.74	0.80
Bernoulli NB	0.55	0.20	0.21	0.24

**Table 12:** Accuracy comparison of proposed system with benchmark system.

ML Algorithms	IoT Accuracy	Std. Accuracy
Support Vector Algorithm	0.75	0.70
Gaussian NB	0.42	0.40
Decision Tree Classifier	0.81	0.80
Random Forest Classifier	0.93	0.90
Bernoulli NB	0.55	0.55

## Data Availability

Data are contained within the article.
